# Reincubation of culture-negative urines for an additional 20 hours does not identify additional UTI cases

**DOI:** 10.1099/jmm.0.001104

**Published:** 2019-12-02

**Authors:** Kaan Kocer, Stefan Zimmermann, Irene Burckhardt

**Affiliations:** ^1^​ Department for Infectious Diseases, University of Heidelberg, Heidelberg, Germany

**Keywords:** urine incubation time, TLA, slower growing organisms

## Abstract

**Introduction:**

The question of whether a single day of incubation is sufficient for urine cultures has been a matter of debate.

**Aim:**

The aim of this study was to investigate the potential benefit of prolonged incubation for initially culture-negative urines.

**Methodology:**

Eight hundred and twelve urine specimens with no growth after incubation for 20 h were incubated for an additional 20 h to detect slower growing uropathogenic organisms.

**Results:**

This study included a considerable number of urine cultures from immunocompromised and/or kidney-transplanted patients. For 99.9 % of the specimens, there was no difference in the interpretation of results.

**Conclusion:**

Twenty hours of incubation did not have any negative effect on the detection of uropathogens.

## Introduction

Incubation time for urine cultures has been a controversial issue, since numerous studies have reported that 24 h incubation time causes the loss of slower growing Gram-positive bacterial species such as *Lactobacillus* spp., *Corynebacterium* spp. or *Gardnerella vaginalis* [1, 2] and yeasts [3]. Furthermore, our studies demonstrated previously that these organisms in particular were isolated more frequently using total laboratory automation (TLA) systems [4]. In addition to this, antibiotic therapy prior to urine culture as a confounding factor may delay or reduce microbial growth [5].

In the present study, we analysed urine samples without significant growth from routine microbiological diagnostics after 20 h. The aim of this study was to investigate the potential benefit of prolonged incubation for initially culture-negative urine.

## Methods

Urine samples (midstream and catheter urines) collected routinely between 1 September 2018 and 2 October 2018 from patients admitted to the Heidelberg University Hospital were examined. Repetitive isolates from the same patient were included.

Ten microlitres of the urine samples was inoculated on Columbia agar with 5 % sheep blood (BD Diagnostics, Heidelberg, Germany) and CPS (bioMérieux, Marcy-l'Étoile, France) plates using the InoqulA module of the BD Kiestra TLA system. After inoculation, the plates were transported automatically to ambient air incubators for CPS plates and 5 % CO_2_ incubators for Columbia agar. All plates were incubated at 35 °C for 20 h before imaging and initial examination by technicians. The cutoff for no growth was 10^2^ colony-forming units (c.f.u.) ml^−1^. All plates interpreted as no growth (<10^2^ c.f.u. ml^−1^) at 20 h were incubated for an additional 20 h (total incubation time of 40 h) prior to a second round of imaging and inspection for growth. Incubation periods of 20 h were chosen for technical reasons, because this was predefined as the imaging incubation time by BD Kiestra. The growth of one or two isolates of possible pathogens at a concentration of ≥10^3^ c.f.u. ml^−1^ was interpreted as positive and species identification was carried out. Quantification was performed by using a validated standardized scheme. In cases where there were three or more different colony morphologies on the plate, the specimens were considered to have been improperly collected (‘contaminated’) and no further identification was performed, regardless of the c.f.u. ml^−1^.

Species identification was performed by matrix-assisted laser desorption/ionization time-of-flight mass spectrometry (MALDI-TOF MS) using a Bruker microflex and the latest Biotyper software and database (Bruker Daltonics, Bremen, Germany).

Laboratory data (age, sex, ward) were obtained from our laboratory information system (SWISSLAB, Roche Diagnostics, Mannheim, Germany). All data were collected retrospectively and anonymized prior to transfer into the study database. Statistical analysis (odds ratio) was performed using STATA13 (StataCorp LP, College Station, TX, USA). To determine the statistical significance of additional growth after 40 h of incubation, a *z*-test for differences between proportions was performed*.* *P*<0.05 was considered to be statistically significant.

## Results

Out of 3517 urine samples sent for microbiological analysis during the study period, 812 were evaluated as no growth at 20 h. These 812 urine samples originated from 627 patients. The mean age and standard deviation of our study population was 58±20 years (range 2 weeks–94 years). Four hundred and forty-seven patients were male (71.3 %) and 180 (28.7 %) were female. Furthermore, 28.7 % (233/812) of the specimens were recovered from patients in the outpatient departments, 37.2 % (302/812) in the normal wards and 34.1 % (277/812) in the intensive care units, for exact distribution (see Table S1, available in the online version of this article).

In 798/812 samples (98.3 %) the reading at 40 h also showed no growth, whereas in 14/812 specimens [1.7 %*;* 95 % confidence interval (CI): 0.7–2.7 %; *z*=3.85; *P*<0.001) from 14 different patient*s* growth was only detected after 40 h. The mean age and standard deviation of these patients was 41.8±19 years (range 12 years–79 years). Three patients were male (21.4 %) and 11 (78.6 %) were female (see Table S2). The exact distribution of specimens according to departments is provided in Table S1. Remarkably, in 13/812 samples (1.6 %) organisms not commonly associated with urinary tract infections (UTIs) or contamination were detected after 40 h (see Table 1). An organism recognized as a uropathogen (* Corynebacterium urealyticum *) was only recovered from 1/812 samples (0.1 %*;* 95 % CI: −0.1–0.3%*; z*=1.00; *P*=0.16) (see Fig. S1), and was collected from a female patient with neuroendocrine tumour of the gallbladder, in very low amounts (10^3^ c.f.u. ml^−1^) [6]. No yeasts were detected with longer incubation. Among 2705 specimens with growth after 20 h of incubation, 32 isolates (1.2 %) showed growth of *Lactobacillus* spp., 5 (0.2 %) *G. vaginalis* and 13 (0.5 %) *Corynebacterium* spp., including 1 (0.04 %) *C. urealyticum* (see Table S3). The leukocyte count of two isolates exceeded 100 µl^−1^, which is also the level suggested by Public Health England to discriminate infection [7], whereas two isolates, including the one with *C. urealyticum *, showed an elevated, though still not significant, leukocyte count (10–100 µl^−1^).

**Table 1.** Patient characteristics of samples with growth after 40 h

Patient	Bacterial species	Age	Sex	Station	Leukocyte count
P1	10^3^ *Lactobacillus* spp.	33	F	OR	0–1
P2	10^3^ *C. glucuronolyticum *	36	M	IM	0
P3	10^3^ more than 2 morphologies	29	F	IM (NTx)	1
P4	10^3^ more than 2 morphologies	66	F	IM (NTx)	7
P5	10^3^ *Lactobacillus* spp.	32	F	EX	na
P6	10^4^ more than 2 morphologies	79	F	RAD	847
P7	10^4^ more than 2 morphologies	28	F	IM (NTx)	0
P8	10^3^ *C. glucuronolyticum *	12	M	SUR	na
P9	10^3^ *C. urealyticum *, 10^3^ CNST	44	F	IM	16
P10	10^4^ *Lactobacillus* spp.	52	F	RAD3	150
P11	10^4^ *G. vaginalis *, 10^3^ *C. glucuronolyticum *	64	M	IM (NTx)	5
P12	10^5^ *Lactobacillus* spp.	55	F	IM (NTx)	0
P13	10^4^ *G. vaginalis *	21	F	EXT	na
P14	10^3^ *Lactobacillus* spp., 10^3^ *Bifidobacterium* spp.	34	F	IM	12

F, female; M, male; IM, internal medicine; SUR, surgery; EX, external clinic; OR, orthopaedics; RAD, radiology; GN, gynaecology; NTx, renal transplantation; na, not available.

Interestingly urine samples from kidney transplant recipients were at a significantly higher risk of having growth after 40 h (*n*=5/78) (OR 5.5; 95 % CI: 1.8–16.9; *P*=0.003).

## Discussion

A small percentage (1.7 %), mostly members of the Gram-positive urogenital microbiota, was missed at the 20 h reading time point. *Lactobacillus* spp. and *Corynebacterium* spp. (except *C. urealyticum *), as part of vaginal flora, are not recognized as pathogenic organisms [8]. As opposed to this, there are numerous reports about the role of *G. vaginalis* as an underestimated cause of UTIs [9]. However, clinical evidence is still lacking. Thus, the specimens with these species were regarded as contaminated by urogenital flora.

Statistical methods confirmed that the increase in the growth of potentially clinically relevant bacteria was statistically not significant. In one sample (0.1 %) an organism associated with UTI was detected by additional incubation [6]. In the literature, *C. urealyticum* has been described as a slow growing organism and hence cultivation up to 48 h is recommended [10]. However, our findings revealed that 20 h incubation rarely misses significant growth of them, because after longer incubation*,* in addition to the single specimen with *C. urealyticum* under standard 20 h incubation*,* only 1 of out of 812 specimens was positive for this uropathogen.

Notably, neither of the two specimens with higher urine leukocyte counts (>100 µl^−1^) belonged to the patient with *C. urealyticum *. In one patient, the elevated leukocyte count may be attributed to the detection of 10^4^ c.f.u. ml^−1^ *E. coli* in urine 1 day prior to the specimen without growth, whereas in the other one, only part of the urogenital flora, namely *Lactobacillus* spp., was identified.

The actual antibiotic therapy of a patient is not reliably reported to our microbiological laboratory, but our study included 17 samples from patients treated in haematology wards. These patients routinely receive antibiotic treatment during immunosuppressive therapy [11]. Remarkably, in these samples no growth was detected with longer incubation. Furthermore, urine samples from kidney transplant recipients were at a significantly higher risk of having growth after 40 h (*n*=5/78) (OR 6.1; 95 % CI: 1.99–18.71; *P*=0.002). Notably, in the five urine cultures recovered from these patients, three were considered to be contaminated due to the presence of multiple species, whereas the remaining two only showed growth of non-recognized UTI agents.

Consequently, the clinical interpretation of the urine culture results was not different for 811 out of 812 samples (99.9 %) after 40 h of incubation. Our data showed that an incubation time of 20 h was clearly reliable for detecting uropathogenic organisms and longer incubation with TLA did not have a significantly positive impact on the recovery of them.

## Supplementary Data

Supplementary material 1
