# Supplementary material for: Reincubation of culture-negative urines for an additional 20 hours does not identify additional UTI cases
Source: J Med Microbiol. 2019 Dec 2;69(1):46–8. doi: 10.1099/jmm.0.001104 (PMC7440675; doi:10.1099/jmm.0.001104)

Table S1 Distribution of analysed urine specimens according to medical specialties and departments

	Medical specialties																				Departments					
	Total		IM		SUR		EX		OR		PD		NE		DE		RAD		GN		ICU		IPD		OPD	
	n	(%)	n	(%)	n	(%)	n	(%)	n	(%)	n	(%)	n	(%)	n	(%)	n	(%)	n	(%)	n	(%)	n	(%)	n	(%)
**All specimens**	812	100	281	34,6	226	27,8	126	15,5	70	8,6	29	3,6	57	7,0	7	0,9	10	1,2	6	0,7	277	34,1	302	37,2	233	28,7
**Specimens without growth after 40h**	798	98,3	273	34,2	225	28,2	124	15,5	69	8,6	29	3,6	57	7,1	7	0,9	8	1,0	6	0,6	277	34,7	297	37,2	224	28,1
**Specimens with growth after 40h**	14	1,7	8	57,1	1	7,1	2	14,3	1	7,1	–	–	–	–	–	–	2	14,3	0	0,0	–	–	5	35,7	9	64,3

Abbreviations: IM: Internal medicine, SUR: Surgery, EX: External clinic, OR: Orthopaedics, PD: Pediatrics, NE: Neurology and Neurosurgery, DE: Dermatology RAD: Radiology, GN: Gynaecology, ICU: Intensive care unit, IPD: Inpatient departments, OPD: Outpatient departments

Table S2 Patient demographics

	**all patients**	**without growth after 40h**	**with growth after 40h**
mean age (years ± SD)	58.01 ± 20	58.37 ± 19.9	41.79 ± 18.99
female (%)	28.71	27.61	78.57

Abbreviations: SD: Standard deviation

Table S3 Slow growing organisms under standard and prolonged incubation

**slow growing organisms**	**culture-positive after 20h of incubation (50 of 2705)**		**culture-negative after 20h and -positive after 40h of incubation (11 of 802)**	
	n	(%)	n	(%)
***Lactobacillus* spp.**	32	1,18	5	0,62
G. vaginalis	5	0,18	2	0,25
Corynebacterium* spp., other than *C. urealyticum	12	0,44	3	0,37
C. urealyticum	1	0,04	1	0,12

Figure S1: Results of 40h incubation

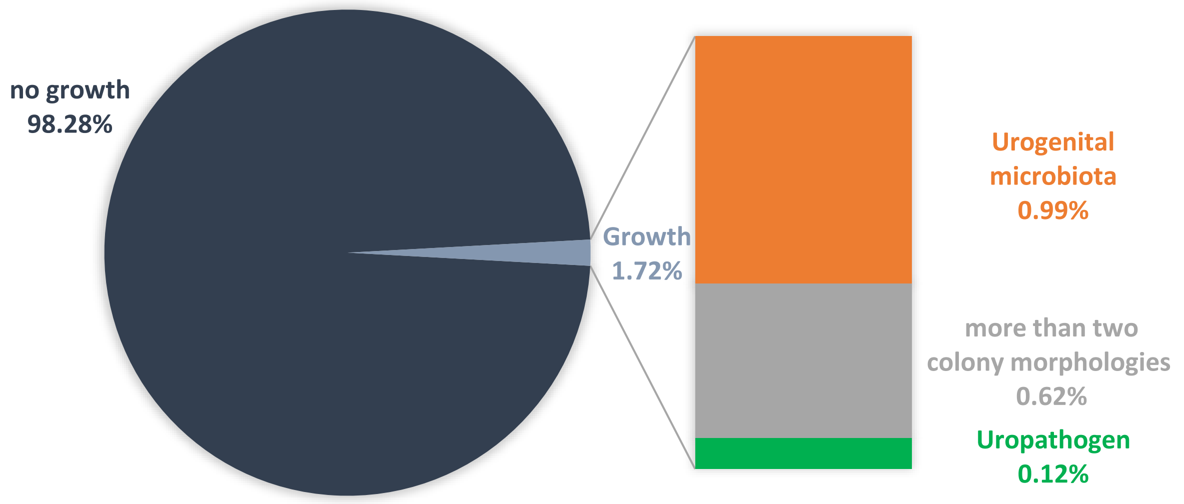

Supplement: Supplementary material 1 [file jmm-69-46-s001.pdf]
